# Effectiveness of Group Problem Management Plus (Group-PM+) for adults affected by humanitarian crises in Nepal: study protocol for a cluster randomized controlled trial

**DOI:** 10.1186/s13063-020-04263-9

**Published:** 2020-04-19

**Authors:** Edith van’t Hof, Manaswi Sangraula, Nagendra P. Luitel, Elizabeth L. Turner, Kedar Marahatta, Mark van Ommeren, Pragya Shrestha, Richard Bryant, Brandon A. Kohrt, Mark J. D. Jordans

**Affiliations:** 1 Department of Mental Health and Substance Use, World Health Organization, Geneva, Switzerland; 2 Transcultural Psychosocial Organization Nepal, Baluwatar, Kathmandu, Nepal; 3 Department of Biostatistics and Bioinformatics and Duke Global Health Institute, Duke University, Durham, NC USA; 4 World Health Organization, Country Office for Nepal, Kathmandu, Nepal; 5 University of New South Wales, Sydney, Australia; 6 Department of Psychiatry and Behavioral Sciences, George Washington University, Washington, DC USA; 7 Centre for Global Mental Health, Institute of Psychiatry, Psychology, and Neurosciences, King’s College London, London, UK

**Keywords:** Group interventions, Cluster randomized controlled trial, Mental health, Humanitarian emergencies, Low- and middle-income countries, Non-specialists, Nepal

## Abstract

**Background:**

Globally, the lack of availability of psychological services for people exposed to adversities has led to the development of a range of scalable psychological interventions with features that enable better scale-up. Problem Management Plus (PM+) is a brief intervention of five sessions that can be delivered by non-specialists. It is designed for people in communities in low- and middle-income countries (LMIC) affected by any kind of adversity. Two recent randomized controlled trials in Pakistan and Kenya demonstrated the effectiveness of individually delivered PM+. A group version of PM+ has been developed to make the intervention more scalable and acceptable. This paper describes the protocol for a cluster randomized controlled trial (c-RCT) on locally adapted Group PM+ in Nepal.

**Methods/design:**

This c-RCT will compare Group PM+ to enhanced usual care (EUC) in participants with high levels of psychological distress recruited from the community. The study is designed as a two-arm, single-blind c-RCT that will be conducted in a community-based setting in Morang, a flood affected district in Eastern Nepal. Randomization will occur at ward level, the smallest administrative level in Nepal, with 72 enrolled wards allocated to Group PM+ or to EUC (ratio 1:1). Group PM+ consists of five approximately 2.5-h sessions, in which participants are taught techniques to manage their stressors and problems, and is delivered by trained and supervised community psychosocial workers (CPSWs). EUC consists of a family meeting with (a) basic information on adversity and mental health, (b) benefits of getting support, (c) information on seeking services from local health facilities with mhGAP-trained staff. The primary outcome measure is levels of individual psychological distress at endline (equivalent to 20 ± 1 weeks after baseline), measured by the General Health Questionnaire (GHQ-12). Secondary outcome measures include levels of functioning, depressive symptoms, post-traumatic stress disorder symptoms, levels of social support, somatic symptoms, and ways of coping. We hypothesize that skills acquired will mediate any impact of the intervention.

**Discussion:**

This c-RCT will contribute to the growing evidence-base for transdiagnostic psychological interventions delivered by non-specialists for people in communities affected by adversity. If Group PM+ is proven effective, the intervention manual will be released for use, giving the opportunity for further adaptation and implementation of the intervention in diverse settings with communities that require better access to psychological interventions.

**Trial registration:**

ClinicalTrials.gov, NCT03747055.

## Background

Globally, the lack of availability of psychological services for people exposed to adversities has led to the development, by the World Health Organization (WHO), of a range of scalable psychological interventions with features that enable better scale-up. The interventions are short of duration and carried out by non-professionals from the communities to make them sustainable and feasible to implement on a broader scale. One of these interventions is Problem Management Plus (PM+) [1, 2]. It has several core features that make the intervention suitable for low-resource settings exposed to adversities. It is a brief intervention of five sessions that can be delivered by non-specialists and is designed for people in communities in low- and middle-income countries (LMIC) affected by any kind of adversity as a transdiagnostic intervention, addressing a range of emotional (e.g., depression, anxiety, stress) problems.

Nepal is a low-income country with a history of humanitarian crises due to conflict, political instability, and natural disasters in the form of earthquakes and monsoon-related floods and landslides. Over 1.6 million people are affected by flooding in Nepal every year. The 2015 earthquake resulted in serious internal displacement, cost the lives of over 8000 people, and injured almost 20,000 people [3]. A large proportion of the population in Nepal is affected by either floods or earthquakes through the loss of livelihood or homes and property. Humanitarian crises and natural disasters cause significant psychological and social suffering to affected populations. Nationwide population-based prevalence data on mental health problems is not available, but various studies suggest high rates of disabling distress [4–8].

There is a large unmet need for mental health care in Nepal, which is especially pronounced given recent and frequent humanitarian crises. There are 0.52 psychologists and 0.36 psychiatrists per 100,000 people [9], mostly working in large cities and inaccessible to those in rural areas. Midwives and community care providers, often working for NGOs, provide primary care in most of Nepal and this system allows for a model of care through non-specialized services as a possible solution to consider [10] .

This paper describes the protocol for a cluster randomized controlled trial (c-RCT) of locally adapted Group PM+ in Nepal. Two randomized controlled trials in Pakistan and Kenya demonstrated the effectiveness of individually delivered PM+ [11, 12]. A group version of the intervention was developed to make PM+ more scalable and acceptable in different contexts. The first trial with Group PM+ in Pakistan showed promising results for women [13, 14] and positive findings from the study described in the current protocol is expected to lead to WHO releasing Group PM+ for global use. This study follows on a feasibility c-RCT conducted in a rural flood-affected region of Nepal [10].

## Methods/design

### Objectives

This study aims to evaluate the effectiveness of the locally adapted Group PM+ intervention in communities affected by adversity in Morang, Nepal. The cluster randomized controlled trial (c-RCT) will compare Group PM+ to enhanced usual care (EUC) in participants with high levels of psychological distress recruited from the community. The primary hypothesis is that at endline (20 ± 1 weeks after baseline for the control arm participants, and 12 + 1–2 weeks after the time of the final group session for the Group PM+ arm participants), people receiving Group PM+ will have lower psychological distress scores, as measured by the General Health Questionnaire (GHQ)-12, compared to people in the EUC control. The secondary hypotheses is that people receiving Group PM+ will also report less severity of depression symptoms, posttraumatic stress disorder (PSTD) symptoms, personalized measures of distress, culture-specific symptoms of psychological distress, and somatic symptoms and higher levels of functioning and social support at the post-treatment assessments. We also hypothesize higher levels of skill use related to the Group PM+ intervention content.

A qualitative component is added to the project with the objective to explore the effectiveness of the intervention and barriers to scale-up of Group PM+ with relevant stakeholders including participants, families, and Group PM+ facilitators.

### Design and setting

The study is designed as a two-arm, single-blind c-RCT that will be conducted in a community-based setting in Morang, a flood-affected district in Eastern Nepal. Outcomes will be measured on participants’ level at baseline and at two additional time points, midline and endline. Midline is 7 weeks after baseline (for the Group PM+ participants, this will be approximately 1 week after concluding the intervention). Endline is 20 ± 1 weeks after baseline for the control arm participants, which is approximately 12 + 1–2 weeks after the time of the final group session for the Group PM+ arm participants. Endline is the primary endpoint for the study.

Administrative levels in Nepal are: (1) provinces; (2) districts; (3) *nagarpalikas* or *gaupalikas* (municipalities or rural municipalities); and (4) wards. Randomization will occur at the ward level, the smallest administrative level in Nepal, with half of 72 enrolled wards receiving Group PM+ and the other half receiving EUC. Importantly, given that the groups of the Group PM+ intervention will be of a single gender (see details below in “Group PM+ intervention” section) and that we do not have resources to enroll more than one group per ward, we will select a subset of 14 of the 72 wards to be those where we enroll male participants and the remaining 58 wards will enroll female participants. This fraction (14/72), close to 20% of all wards, was selected to reflect the anticipated uptake of services, which was expected to be lower in this region than in studies conducted by our team in other regions [15, 16]. Further, we note that the selection of 14 wards will not be random but instead those 14 wards will be selected to be 14 wards that are close together and that are, nevertheless, representative of the types of wards in the study region. More specifically, we selected these 14 “male” wards close together so that we can best use resources of the male personnel trained to deliver to the Group PM+ intervention. Because of the sub-selection of “male” and “female” wards, randomization will be stratified by gender and will account for several other baseline cluster-level covariates using restricted randomization (see details below in the “Randomization and sample size” section).

The c-RCT is the design of choice when an intervention is group-based and when the population is expected to receive clinical and community services according to their location (i.e., ward) of residence. An alternative design is an individually randomized group treatment trial (IRGT) in which individuals, rather than clusters, are randomized [17, 18]. An IRGT design is typically expected to have greater power than a c-RCT for the same number of enrolled individuals and same degree of outcome clustering. However, such a design would not be suitable given concerns about contamination of the intervention within wards had there been both Group PM+ and EUC participants in each ward.

**Fig. 1:**
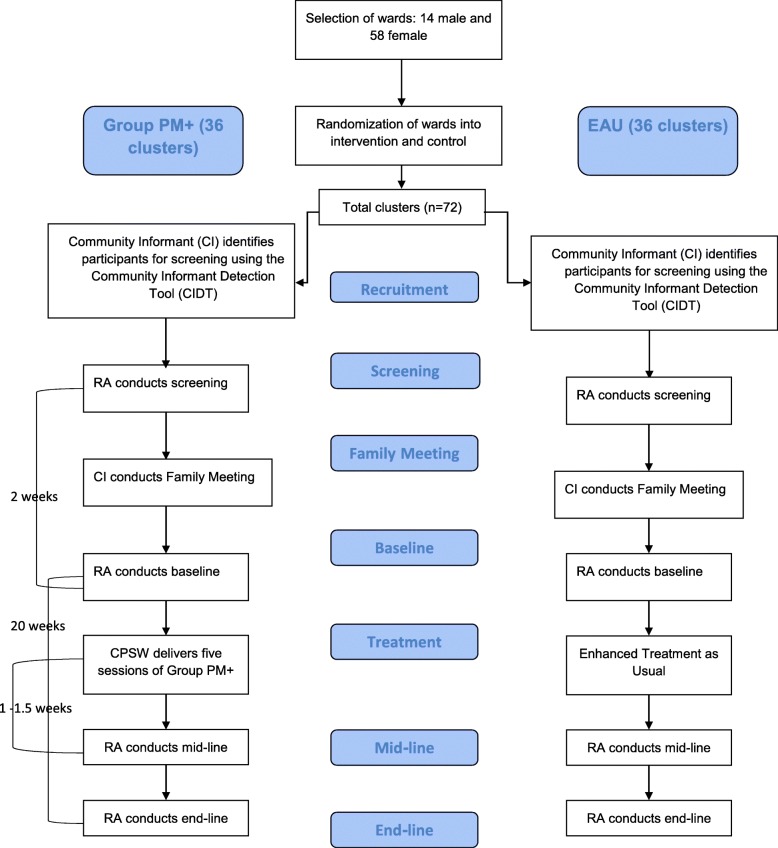
Consort flow chart

Additional enrolment strategies will be employed to minimize the risk of contamination. Specifically, given that some wards will be contiguous with each other, before participant recruitment begins, we will map the area and specify a localized area within each ward from which we will seek to recruit participants. The locations within the wards will be selected so that recruited participants from each ward are geographically far from those recruited in neighboring wards to minimize the chance that participants from different wards (i.e., from different clusters) interact with each other. Such a strategy will be used to conserve independence of clusters and to avoid contamination of EUC clusters with information from the Group PM+ intervention. Figure 1 gives an overview of the design.

This community-based study is being conducted in five municipalities and three rural municipalities that together encompass 72 wards within Morang, a densely populated district in the Eastern *terai* (lowland) region of Nepal. The selected areas have a diverse population with over 20 castes and ethnicities, including Tharu, Brahman/Chhetri, Yadav, and Rai. The national language of Nepali is spoken by the majority of inhabitants. Morang is flood-affected annually and in 2017 it was estimated that over 19,000 people were displaced and over 12,000 homes were partially damaged due to the natural disaster [10]. Three Primary Health Care Centers (PHCCs) within the selected areas provide basic healthcare and have an attending health worker trained in the WHO mental health Gap Action Program (mhGAP) and will be used for EUC referral.

### Study arms

#### Group PM+ intervention

Problem Management Plus (PM+) is a WHO trans-diagnostic psychological intervention that is delivered by trained non-specialist lay-providers in five sessions to adults impaired by distress [1, 2]. The manual comprises of the following evidence-based techniques: (a) problem solving, (b) stress management, (c) behavioral activation, and (d) accessing social support.

The Group PM+ intervention consists of five 2.5- to 3-h sessions in which participants are taught techniques to manage their stressors and problems. Table 1 gives an overview of the content of the five sessions. The aim is to have six to eight participants per group, with separate groups for men and women and with gender-matched facilitators. Information on seeking services from local health facilities with mhGAP-trained health care staff trained in providing mental health care and/or psychosocial support is provided to the Group PM+ participants as well as to the EUC participants.

**Table 1:** Mechanisms of action of PM+ intervention

PM+ mechanisms of action	Description of mechanism	Implementation of mechanism
*Stress management*	Participants learn deep breathing. They are encouraged to incorporate this mechanism into daily life (i.e., when doing housework, walking, etc.). Grounding techniques are incorporated to bring participants back to the present	Session 1
*Behavioral activation*	Participants review the inactivity cycle. They choose a small activity that they enjoy doing (i.e., making and drinking tea, meeting a friend etc.) and create a detailed plan about when and how to conduct this activity as a first step in breaking the inactivity cycle	Session 2
*Managing problems*	Participants learn which of their problems are solvable and which are unsolvable. One problem is chosen amongst the solvable problems and participants brainstorm tangible solutions, then create manageable steps to accomplish their goals	Session 3
*Strengthening social support*	Participants learn to recognize who amongst their family and friends are existing and potential sources of support and how best to strengthen connections with them. Social network mapping activities are incorporated into this mechanism	Session 4

The first four sessions of PM+ each addresses a specific mechanism of action. The fifth and last session is a review of the mechanisms of actions learned in the previous sessions

Community psychosocial workers (CPSWs) are trained as Group PM+ facilitators [10]. CPSWs are a cadre of community health workers that have a long track record in providing psychosocial support in Nepal [19]. For this study individuals from the community will be recruited to become new CPSWs. Fifteen local community women and men who have completed higher secondary school (equivalent of 12th grade education) from the study region will be selected based on their basic communication skills as reflected through the interviews, management and organization skills, interest and motivation to serve community people, and commitment to work in the given time. They are then given a 10-day basic CPSW training, with a standard curriculum developed by TPO Nepal. The CPSW training includes an overview of psychosocial concepts, cause and effects of psychosocial issues, basic communication skills, common mental health problems in communities, group facilitation skills, and psychoeducation. Competency is evaluated before and after the CPSW training with a standardized role play assessment tool (ENACT) that has been developed in Nepal and used for non-specialists in humanitarian settings [20].

The CPSW training is followed by a 10-day Group PM+ training using the adapted manual and other intervention materials. Group PM+ is named *Khulla Man* (“open heart-mind” in Nepali), which is consistent with Nepali ethnopsychological models of distress, trauma, and recovery. The Group PM+ training includes learning about the impact of adversity on mental health, basic counseling skills, how to deliver the content of the Group PM+ manual, group management skills and self-care. Competency is assessed with ENACT again at the conclusion of the PM+ training, and fidelity is assessed with a PM+ specific checklist.

After completing PM+ training, three rounds of practice sessions will be completed by each CPSW in an adjoining district that is not a part of the study area. Competency assessments and supervision will be conducted during these practice sessions. Based on ENACT pre and post scores, clinical judgment during the PM+ practice sessions, assessments using the fidelity sheet, and the PM+ competency criteria, 12 CPSWs (ten female and two male) out of 15 will be selected. In regards to ENACT, the CPSW who scores the lowest points, i.e., 1 (Need improvement) for each item, will be removed from the study.

Three types of supervision will be provided by TPO Nepal supervisors for PM+ providers while running the PM_ groups. Firstly, face-to face group supervision will be provided in the office twice a week for Group PM+ facilitators. Secondly, there is on-site supervision, in which a supervisor will sit in and observe at least two sessions per PM+ group. Fidelity and competency assessments will be conducted during these sessions to verify the delivery of Group PM+ to participants. Intervention fidelity is monitored through independent observations of 10–15% of sessions of each facilitator against tailored checklists. Fidelity and competency tools will be used and direct feedback will be given to PM+ facilitators leading the group. These sessions will also be audio recorded and reviewed in the in-office supervision sessions. Lastly, individual supervision sessions between the supervisors and Group PM+ facilitators will be conducted as needed. Supervision sessions will be documented using standard supervision forms and facilitators will discuss any reoccurring or unique challenges and successes during the sessions with the supervisors.

Facilitators are supported by assistants called “Group PM+ helpers” who receive a basic 1-day training on assisting Group PM+ delivery and participate alongside CPSWs in practice PM+ groups. They help with the logistics and organizational aspects of the group sessions, such as reminding participants when sessions take place, reminding those that do not show up for the sessions, and providing child care. Additional tools such as calendars, session cards, and reminders, all developed specifically for the Nepal implementation of Group PM+, are used to increase retention of the material and attrition by participants.

#### Enhanced usual care

In rural regions of Nepal, care-as-usual for most people with mental health problems until recently consisted of no psychological or psychiatric treatment in local health facilities. People with severe mental conditions would often, after a long delay between onset of symptoms, be taken to tertiary psychiatric services in the Kathmandu valley, or other urban settings with psychiatric services, by family members [21]. The Programme for Improving Mental Health Care (PRIME) has been implemented in Chitwan district, in southern Nepal, and has implemented and evaluated the WHO mental health Gap Action Programme (mhGAP) Intervention Guide since 2012 [22, 23]. The mhGAP *Humanitarian* Intervention Guide [24] was contextualized for Nepal after the 2015 earthquakes and Nepali primary care workers in many districts, including Morang, have since been trained using mhGAP. Both the EUC and intervention arm will receive a referral to a mhGAP trained primary health care worker providing treatment when needed (e.g., severe psychiatric disorder or suicidality) .

Participants in the EUC control clusters will receive a time-restricted (between 30 and 45 min) family meeting conducted by local Community Informants (CIs) that will consist of: (a) basic information on adversity and mental health, (b) benefits of getting support, (c) information on seeking services from local health facilities with mhGAP-trained health care staff trained in providing mental health care and/or psychosocial support [10]. The mhGAP training that these health care staff received consists of a 6-day training, focusing on a selected number of mental disorders including common mental disorders, including an additional module on anxiety disorders (excluding PTSD). This family meeting will be conducted with family members of the participant or the participant only based on participants’ preferences. Both arms will receive the same family meeting format and referral information to primary care-based treatment.

### Randomization

The unit of randomization is the ward (i.e., the cluster), as this is the smallest unit of administration in Nepal. This unit was selected to ensure a sufficient number of clusters, as there are only 17 municipalities/villages in the district, which would be the next possible level of randomization. Municipalities with mainly non-Nepali speaking inhabitants will be excluded. A total of 72 wards will be selected for participation with a target sample size of eight participants enrolled per ward (see rationale below in the “Sample size justification” section). Then, for the 36 wards randomly allocated to Group PM+, a single group of eight participants will be formed in each ward. As indicated above, of these 72 wards, 14 will be selected as “male” wards and 58 as “female” wards to reflect differences in uptake of services by males compared to uptake by females, as observed in earlier studies conducted by our team (see above). As such, the overall estimated intervention effect will reflect such a 1:4 ratio of males:females should the intervention be scaled up more broadly. Furthermore, as noted above, we will not take a random sample of 14 wards as “male” since it is important that the selected wards are such that whichever seven are randomly allocated to Group PM+ are sufficiently close in proximity so that it will be reasonably straightforward for two male CPSWs to lead the seven male Group PM+ groups (i.e., one in each of the “male” Group PM wards).

Restricted randomization will be used. Specifically, we will first use stratification by “ward gender” (i.e., randomization separately within 14 “male” wards and within 58 “female” wards). Then, within each “ward gender”, we will use covariate constrained randomization to account for three baseline cluster-level covariates that are expected to be related to participant outcomes and for which it is important for us to achieve balance between the two study arms. Those three covariates, all defined as binary, are: (1) access to mental health services (high or less than 1 h to reach nearest PHCC vs. low or less than 1 h to reach nearest PHCC); (2) disaster risk (high or landslides or flooding in the last 3 years vs. low-to-moderate or minimal landslides or flooding in the last 3 years); and (3) rural/urban status (rural defined as wards that do not touch a major highway, majority of homes made of wood/straw/mud, and no local markets and urban defined as wards close to highways, majority of homes made of concrete and access to local markets). Covariate constrained randomization is a generalized form of stratification which can be used to simultaneously balance on multiple baseline covariates without the need to formally define strata based on the cross-classification of those covariates [25]. In practice, in order to perform covariate-constrained randomization within the two strata defined by the 14 “male” wards and the 58 “female” wards, we will separately implement covariate constrained randomization in Stata software (version 14 [26]) using the *cvcrand* procedure [27]. Randomization will be performed in advance of enrolment of participants and will be conducted by the study statistician who does not know the study region. The statistician will use a simple data set with only the ward codes and three relevant covariates to ensure that there is no room for bias in the implementation. Moreover, a seed will be set so that the implementation is reproducible in Stata statistical software.

### Sample size justification

The c-RCT was designed to have at least 90% power to detect moderate effect sizes of 0.46 for the primary outcome of individual psychological distress, measured by the GHQ-12 questionnaire (see details below in the “Outcome Measures” section) at the primary time point of follow-up 20 ± 1 weeks after baseline for the control arm participants, and 12 + 1–2 weeks after the time of the final group session for the Group PM+ arm participants (i.e., endline). An effect size of 0.46 would correspond to between-arm differences of 3.2 units in mean GHQ-12 for an overall standard deviation of 7 units, a conservative assumption based on data from our pilot c-RCT [28]. Power was calculated in R software (version 3.4.2) by programming a standard calculation for a comparison of two means in a c-RCT with 72 clusters assuming a two-tailed 5% significance level [29]. It was additionally assumed that eight participants would be enrolled in each ward, and that up to two participants per ward would drop out before outcomes were measured (a conservative assumption for the purposes of the power calculation). Clustering of outcomes by ward was assumed to be relatively large with an interclass correlation coefficient (ICC) of 0.2 based on baseline data from a cohort study in the Chitwan district used in the PRIME study [22]. Although clustering in the EUC wards is anticipated to be lower than the assumed 0.2 in the Group PM+ wards because EUC participants will not meet in groups, we conservatively assume the same levels in both arms for the purposes of the power calculation.

### Participants

People living in the 72 selected wards in Morang district are eligible to participate when they are over 18 years old and understand and speak Nepali. Inclusion criteria to be eligible for the trial are (1) answering affirmative to the heart-mind screener and for functional impairment [30] and (2) scoring above 16 on the WHO Disability Assessment Schedule for functional impairment (WHODAS) [31]. The heart-mind screener is locally developed (sensitivity of 0.94) and will be used to determine the acceptability of local idioms of distress and impairment due to these problems [30]. The WHODAS is a generic instrument assessing health and disability that can be used with adult populations across cultures. Additionally, only males will be eligible for enrolment in the 14 “male” wards and, similarly, only females will be eligible for enrolment in the 58 “female” wards. Exclusion criteria for participation in the trial are (1) presence of a severe mental disorder (e.g., psychosis) or cognitive impairment identified by a score above 2 on an adapted version of the WHO Ten Questions Screen (TQS) for disability detection [32] and (2) alcohol use disorder (score > 16 on the alcohol use disorders identification test (AUDIT)).

Imminent risk of suicide will be determined through a structured screening questionnaire. Persons with current suicidal ideation and suicide plans or recent attempts will be referred immediately to a psychosocial counselor but will not be excluded from participating in the study. Observable symptoms of psychosis and severe cognitive impairment will be assessed using an observation checklist. Four items are included to examine the client’s ability to comprehend questions and follow basic instructions, and the degree to which the client can communicate with the assessor. A positive response above 2 on any of these behavioral items is an indication for exclusion and is discussed with a supervisory team. Alcohol dependency will be assessed by the alcohol use disorders identification test (AUDIT) [33]. According to WHO’s guidelines for AUDIT use in primary care, people that score below 16 can benefit from simple advice [34]. Those with a score of 16 or over would benefit the most from advice plus brief counseling and continued monitoring and, therefore, those that score 16 or above on the AUDIT will be excluded from the study and referred to a nearby mhGAP-trained health professional [10] .

### Procedures

Each ward of participating municipalities in Morang district will have one community informant (CI) who will conduct recruitment through the use of the Community Informant Detection Tool (CIDT) and community sensitization activities. CIs are often Female Community Health Volunteers (FCHVs), mothers’ group members, or social mobilizers within their respective communities. CIs will, as much as possible, also be gender-matched for the “gender” of their wards. CIs from intervention and control wards will be trained separately to maintain blinding. Control ward CIs will not be given any information on Group PM+ or any other information about the existence of an intervention arm. Intervention CIs will additionally be given a 1-day training to become Group PM+ ‘helpers’ for the sessions.

The CIs will be trained on the CIDT to identity people with common mental disorders in the community. The CIDT is a pro-active case detection approach aimed to increase help seeking using a vignette-based tool designed for the ease of use by lay people. It has been developed and tested in Nepal [35], with positive results on the positive predictive value (0.68) and increasing the utilization of mental health services [36]. A general distress CIDT version had been adapted for this trial [28], which includes gender-matched vignettes for the “gender” of the wards.

After the community informant identifies a person in the community who matches the symptoms described in the vignettes, they will be asked if they would like support for their problems. If so, the research assistant (RA) will then conduct the consent and screening procedures.

People who are identified as meeting the exclusion criteria initially by the RAs will be referred to health workers trained in mhGAP, hospitals with psychiatric services, or counselors. People that meet the inclusion criteria for the study, in both the intervention and control wards, will receive a visit from the CI for a family meeting. Based on the preference of the participants, this can either be with or without their family. After the family meeting, RAs will conduct the baseline assessment with enrolled participants. Once baseline is completed, only those in the intervention group will be contacted by CPSWs to inform them about Group PM+. After all participants in an intervention ward have been contacted by the CPSW, Group PM+ sessions will start.

### Informed consent

The consent procedures consist of two steps, first informed consent for screening and then informed consent for participation in the Group PM+ trial [10] . After identification by the CI, potential eligible people will be approached by the research assistant for informed consent for screening. If a participant screens positive, the CI will give more information about the research project and will conduct the full trial informed consent during the family meeting.

All respondents who decide to participate will provide written consent, if possible. Full information on the study will be provided in local, lay Nepali language before obtaining consent from each participant. Given high rates of illiteracy, the consent form will be read to all participants. After providing verbal consent, literate participants will be asked to acknowledge the process with a signature. For illiterate participants, verbal consent or adding a symbol or sign will be sufficient. We will make sure that potential participants fully understand what participation entails and that they, at any time and without any consequences, can withdraw their consent without having to give an explanation.. Participants will be made aware that refusal to participate will not have an impact on any type of support they receive outside the study. For the qualitative interviews, separate written informed consent will be taken at the time of the interview.

### Outcome measures

#### Primary outcomes

The primary outcome is levels of individual psychological distress, measured by the GHQ-12 [37, 38], at endline, 20 ± 1 weeks after baseline for the control arm participants, and 12 + 1–2 weeks after the time of the final group session for the Group PM+ arm participants. The GHQ-12 consists of 12 questions that are scored on a four-point Likert scale ranging from 0 to 3, with higher total scores representing higher levels of distress. The GHQ-12 has been translated and clinically validated in Nepal (cut-off 1/2, sensitivity 85.6%, specificity 75.8%, positive predictive value (PPV) 86.7%, negative predictive value (NPV) 84%) [39].

#### Secondary outcomes

Secondary outcomes include levels of depressive symptoms measured by the Primary Health Questionnaire (PHQ) [40]; general functioning measured with the WHO Disability Assessment Scale (WHODAS) [31]; post-traumatic stress disorder (PTSD) symptoms measured by the Post-traumatic stress disorder Check List (PCL-5) [41]; levels of perceived social support measured by the Multi-dimensional Scale of Perceived Social Support (MSPSS) [42]; and the Somatic Symptom Scale 8 (SSS-8) [43]. Please see Table 2 for an overview of the different measures on different time-points (Table 3).

**Table 2:** Quantitative outcome measures

Construct	Instrument	Description	Assessment time periods			
			Enrollment(−***t***_***1***_)	Baseline (t_0_)	Midline (t_1_)	Endline (t_2_)
***Screening (participants)***						
Daily functioning	*WHODAS*	Participants rate their ability to engage in daily activities	X			
General psychological Distress	*Heart-mind*	Participants note if they have had any *“man ko samasya”* or heart-mind problems recently	X			
Alcohol use disorder	*Alcohol Use Disorders Identification Test (AUDIT)*	Participants rate alcohol use and associated behavior, as well as daily ethanol consumption	X			
Suicidality	*Suicidality*	Participants rate if they have recently had suicidal thoughts, ideation, and plans	X			
***Primary outcome (Participants)***						
General psychological distress	*General Health Questionnaire (GHQ-12)*	Participants measure their general psychological distress		X	X	X
***Secondary outcomes (participants)***						
Depression symptoms	*Depression symptoms (PHQ)*	Participants rate depression symptoms over past two weeks		X	X	X
Daily functioning	*WHODAS*	Participants rate their ability to engage in daily activities		X	X	X
Post-traumatic stress symptoms	*PTSD Checklist for DSM5 (PCL-5)*	Participants rate their post-traumatic stress symptoms on a scale		X	X	X
Perceived social support	*Multidimensional Scale of Perceived Social Support (MSPSS)*	Participants assess their own connectedness with close family, friends and other forms of support		X	X	X
Somatic symptoms	*Somatic Symptom Scale-8 (SSS-8)*	Participants rate how much they have been bothered by somatic symptoms		X	X	X
General psychological distress	*Heart-mind*	Participants note if they have had any *“man ko samasya”* or heart-mind problems recently			X	X
**Additional measures of mechanisms and potential moderators**						
Ways of coping	*Reducing Tension Checklist (RTC)*	Participants assess their own behavioral and psychosocial skills related to coping		X	X	X
Traumatic events	*Traumatic Events Inventory (TEI)*	Participants rate if they have been exposed to certain traumatic events throughout their lifetime		X		X

**Table 3:**
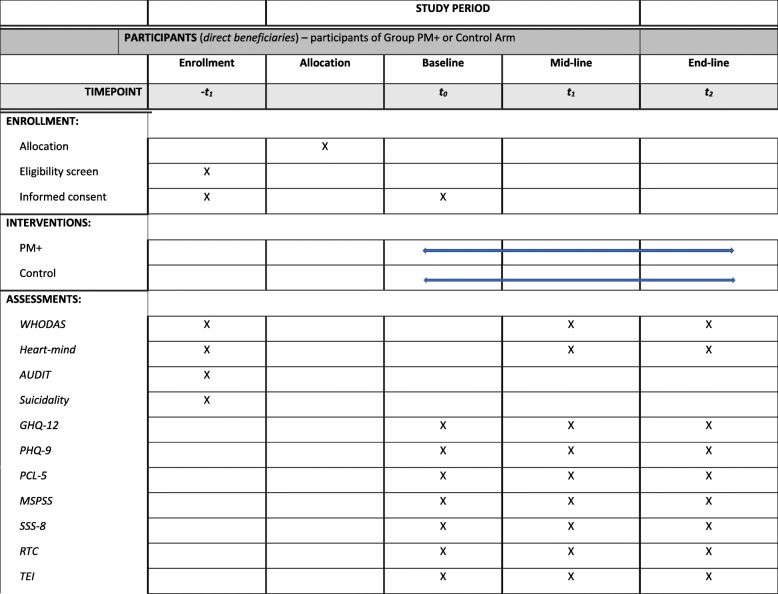
Schedule of enrollment, interventions, and assessments for Group PM+

The WHO Disability Assessment Scale (WHODAS) is a generic instrument assessing health and disability in adults. It assesses difficulties that people are experiencing during the last 30 days, due to their illness, across six domains of functioning (cognition, mobility, self-care, getting along, life activities, and participation). Difficulties are scored on a five-point Likert scale of: not at all difficult, a little difficult, sometimes difficult, very difficult, or always difficult. The WHODAS can be used with all diseases and across cultures. The scale has been previously used in Nepal and has a good internal consistency between items (α = 0.90) and validity with multiple mental health measures for depression (*r* = 0.616, *p* < 0.001), anxiety (*r* = 0.624, *p* < 0.001), and PTSD (*r* = 0.499, *p* < 0.001) [4, 44].

The Patient Health Questionnaire (PHQ-9) is a ten-item instrument measuring symptom depression [40]. It has been translated and clinically validated in a primary care population in Chitwan, Nepal: the validated cut-off score of ≥ 10 (sensitivity = 0.94, specificity = 0.80, PPV = 0.42, NPV = 0.99, positive likelihood ratio = 4.62, and negative likelihood ratio = 0.07) [30].

The original Post-traumatic stress disorder Check List (PCL)-5 is a 20-item checklist corresponding with the 20 DSM IV PTSD symptoms. To diminish the burden of questionnaires administered by participants in this study, the eight-item version will be used. This was shown in a recent study to have comparable diagnostic utility to the 20-item PCL-5 [45] and has been used in Nepal and will be used in this study to diminish the burden of questionnaires administered by participants [46].

The Multidimensional Scale of Perceived Social Support (MSPSS) [42] is a self-rating tool of perceived social support from three categories of support: family, friends, and significant other. It has been locally adapted [47] and validated to use in Nepal [48]. The MSPSS consists of 12 questions that are rated on a five-point Likert scale ranging from 1 (“very strongly disagree”) to 5 (“very strongly agree”). Higher scores indicate higher perceived levels of social support.

The Somatic Symptom Scale (SSS) is an eight-item patient-reported outcome measure of somatic symptom burden [43] that has been translated and adapted using a standard cross-cultural approach [49].

#### Other measures and further data

Competency and fidelity will be assessed with a modified version of the Enhancing Assessment of Common Therapeutic Factors (ENACT) tool tailored for Group PM+ [50]. The ENACT scale is an 18-item assessment for common factors in psychological treatments that can be used by non-specialists in different settings.

Demographic characteristics of participants will be recorded at baseline, including age, years of education, occupation, and living situation. Traumatic events will also be assessed with the Traumatic Events Inventory (TEI), an 11-item assessment of traumatic exposure associated with poor mental health outcomes [51]. The TEI has previously been used in Nepal [52] . A natural disaster questionnaire has also been developed for this trial. This consists of five questions on whether participants were affected by floods, earthquakes, landslides, fires, or other natural disasters in the last 5 years. Participants will be asked if their property was damaged and if they themselves or any relatives and friends were hurt by such natural disasters. Behavioral and psychosocial skills related to coping with emotional distress will be assessed with the Reducing Tension Checklist, which contains a 12-item assessment of behavioral and psychological skills to evaluate skill acquisition of PM+ skills with one free response question based on the PSYCHLOPS [51, 52]. It has been adapted based on PM+ content and findings in phase 1 of the project [28].

During PM+ sessions the Subjective Units of Distress Scale (SUDS) will be used. The SUDS, a scale of 0 to 10 for measuring the subjective intensity of disturbance or distress currently experienced by an individual [53], will be used for each participant during the second to fifth PM+ sessions. The scale has been previously used in Nepal [54].

### Masking

In this project, research assistants administering all interviews, community informants, research supervisors, and study statisticians will be blinded. The intervention does not allow for the intervention facilitators and participants to be blind to treatment allocation. Blinding of assessors will be ensured by minimizing the chance of contact between assessors and facilitators and having two separate offices for the research and clinical staff. Assessors will also prompt participants not to share any information on the type of treatment that they receive and explain that they are not supposed to know. After each assessment, assessors will be asked to indicate what treatment they think each participant will or has received (e.g., medication, one-on-one counseling, group counseling, referral, etc.). This will provide some data on the amount of unblinding that might occur in the RCT. Furthermore, each of the research assistants sign a contract in which they agree to not share any details of the study with others.

**Fig. 2:**
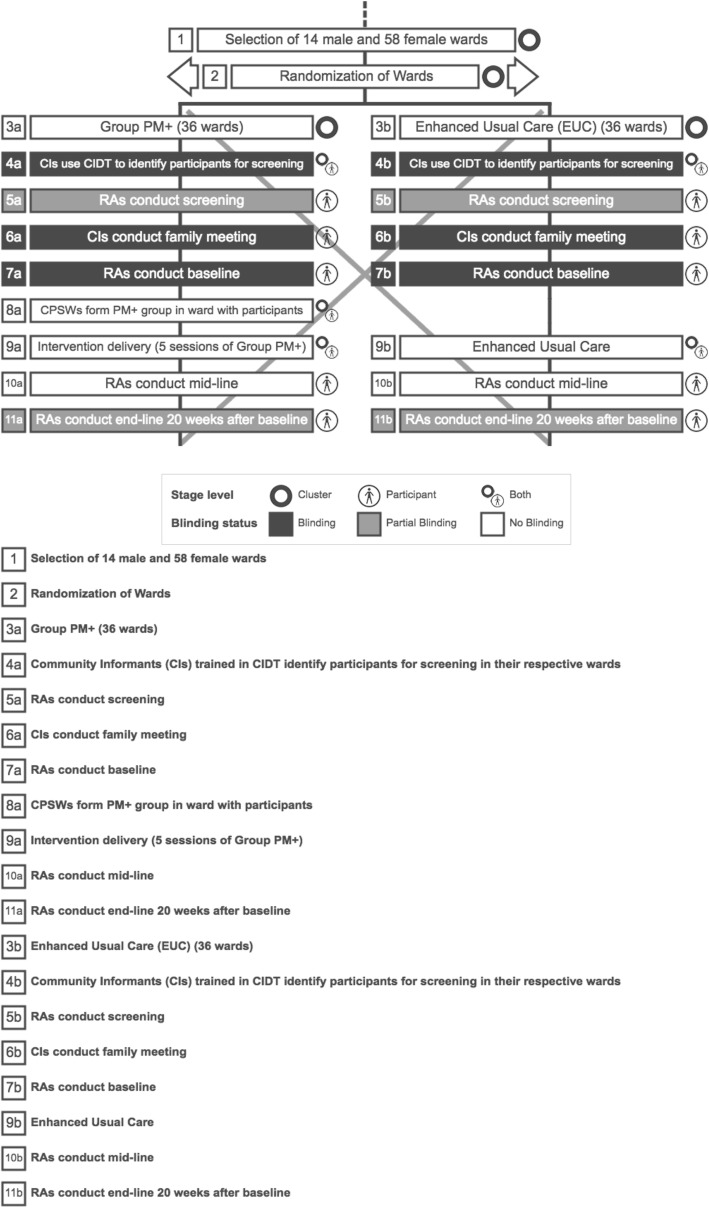
Cluster Timeline

Given the challenges of blinding in c-RCTs and the concerns about the potential for selection bias given that participant recruitment occurs after randomization of the wards in which the participants reside [55], we have used the “timeline cluster” to visualize procedures in relation to blinding and participant recruitment [56]. Specifically, we generated Fig. 2 using an online open-access tool developed by the “timeline cluster” authors [56]. This figure provides additional details to complement the overall study flow chart (Fig. 1), including information on whether a specific stage of the process pertains to clusters, to participants, or to both. The dark boxes indicate stages in the procedure when both participants, and the study personnel who will interact with those participants, will be blinded to which arm the cluster has been allocated to. We will use a design so that study participants are recruited by trained RAs who do not know which arm the ward (cluster) has been assigned to (see up to stage 7 in each arm, Fig. 2). During service delivery (stages 8–9a in Group PM+ and stage 9b in EUC), participants cannot be blinded to study arm. As noted above, however, we have designed the midline and endline data collection procedures so as to try to ensure that the RAs conducting the interviews are blinded to study arm (stages 10–11 in both arms), which is indicated by the light grey shading (i.e., indicating partial blinding because the participants are no longer blinded at this stage). Importantly, when commencing the interview, the RA will emphasize to the participants how important it is that the participants do not reveal details about what kind of services they have received. We recognize that, within a specific ward, if an RA is inadvertently unblinded while conducting the interview with a participant before the final interview in that ward (i.e., before interviewing the eighth of the eight enrolled participants), that RA would therefore be unblinded for the interviews of remaining participants. We will record data as to whether such unblinding occurred and therefore will be able to report on any threats to data validity. And, even in such a case, the RAs receive rigorous and comprehensive training on procedures to objectively record responses to our instruments and measures and, therefore, we expect to be able to mitigate any potential for measurement bias that could arise as a result of unblinding.

### Data management

The research team will keep the identifying key, linking the name to code numbers, in a secure location and only the study principal investigators (PI) of the study will have access to primary data. Research assistants will not enter any personally identifying details into the data set. Data will be collected using a password-protected table, from where data will be synchronized and uploaded in the Open Data Kit (ODK), saved on a private server, and transferred to a data-analytic computer program (STATA) without the identifying key. The site PI will conduct quality assurance checks on data collected by the research assistants on the tablet.

The data collected with other means, like qualitative data and other documentation (e.g., supervision forms and training reports), will be safely stored in locked cabinets at the site office. The qualitative data will be fully anonymized and coded and will not contain any identifying information. Results of this project will be published regardless of being negative or positive and submitted to peer-reviewed scientific journals.

### Data analyses plan

#### Statistical analyses

Analysis of quantitative outcomes, including the primary outcome of GHQ-12, will adopt the intention-to-treat approach whereby all participants will be analyzed according to the arm to which their ward was randomized. That is, even if intervention arm participants did not attend all Group PM+ sessions, the primary analysis will include them in the Group PM+ arm. The linear mixed effects modeling approach will be used to model participant-level score outcomes. More specifically, the two follow-up time points (midline and endline) will be analyzed within the same model. The following design variables will be included as fixed effects: arm, time (an indicator for the follow-up time-point), the arm-by-time interaction (to allow for different intervention effects at each of the two follow-up time-points), ward gender (to account for the stratified design), and the three covariates used in the constrained randomization procedure (i.e., access to mental health services, disaster risk, and rural/urban status). To increase statistical power, each participant’s baseline measure of the outcome will be adjusted for as a fixed effect [57]. To account for clustering by ward, a random intercept will be included for which the degree of clustering is allowed to differ for intervention and control arm clusters. Due to the repeated follow-up measurements on participants, a random intercept will be included for participant. In the event that baseline outcome data are missing, we will use a constrained longitudinal analysis approach whereby the baseline measure is also modeled as an outcome (rather than a covariate) and the baseline mean level is constrained to be equal between arms [57]. In this case, we will allow for changing correlation of outcomes over time by additionally including a random slope for each individual or by using an unstructured residual correlation matrix. For score outcomes for which the assumptions of the linear mixed model are violated, we will transform the outcomes (e.g., log-transformation) or adopt a bootstrap approach to estimate confidence intervals. Binary outcomes will be analyzed within the generalized estimating equations framework. Specifically, we will use the modified Poisson approach [58] assuming a Poisson outcome distribution, with an exchangeable working correlation matrix and robust standard errors to account for the outcome model misspecification (i.e., Poisson instead of binomial). Such an approach has been shown to be preferable to a binomial regression model for clustered outcome data [58]. A log link will be used to obtain risk ratios and an identity link to obtain risk differences and the mean model will include the same terms as the models for the continuous outcomes.

Additional supportive analyses will test robustness to missing outcomes, to baseline covariate imbalance, and to the combination of both. Specifically, the supportive analyses will include the following three approaches: (1) analyses that account for any baseline covariates that are predictive of missing outcomes, (2) analyses that account for any baseline covariates identified to be imbalanced between treatment arms, and (3) analyses that combine both approaches (1, 2), i.e., that account for all baseline covariates identified to be predictive of missing outcomes or to be imbalanced. For approach (1) to assess robustness to missing outcome patterns, if the probability of missingness is only related to the baseline covariates in the model, then these adjusted analyses will provide valid estimates of the intervention effect having accounted for the missing data patterns.

Sub-group analyses will assess whether there are differing intervention effects according to the following variables: gender and baseline depressive symptoms. To do so, the model will include an indicator for the sub-group variable and interactions between that indicator and intervention arm and time-point. Baseline depressive symptoms will be included in the model as a binary variable indicating whether the participant met the cutoff score for depressive disorder, specifically a baseline PHQ-9 score of 10. These analyses are exploratory in nature as the study is not powered to detect such effects. Adherence in the intervention arm will be quantified through the number of sessions attended. Similarly, within the intervention arm, we will examine potential differences in intervention due to different facilitators. To do so, we will analyze outcomes in intervention arm only and see its relationship with facilitator. Likewise, within the intervention arm, we will examine whether estimated outcomes are different for those who completed all five sessions vs. those who completed fewer sessions.

We hypothesize that skills acquired will mediate any impact of the intervention. To this end, we will perform a mediation analysis within the framework outlined by Zhang et al. [59] that accounts for the multilevel (i.e., clustered) data structure. We will use the midline measure of the *Reducing Tension Checklist* as the mediating variable and the endline time-point for outcomes of interest. We note two important features of this analysis: (1) we have selected the midline measure for the hypothesized mediating variable to ensure that it precedes the outcome measure in time in order to be able to make stronger causal claims than we would were the mediator and outcome measured at the same point in time, and (2) we will ensure that potential confounders of the mediator–outcome relationship are accounted for in the analysis.

### Qualitative evaluation

Semi-structured interviews will be conducted with a subsample of Group PM+ participants (equal number of completers and non-completers); Group PM+ facilitators; control arm participants; research assistants; family members of Group PM+ participants (equal number of intervention completers and non-completers); community informants; and local decision makers. The interviews will be conducted by trained interviewers that are familiar with the key principles of qualitative interviewing. Interviews will follow a semi-structured topic guide that address themes around barriers and facilitators in implementing PM+, satisfaction with the intervention, barriers and facilitators to adherence, and barriers and facilitators to scale up and integrating Group PM+ into other services.

All interviews will follow the same process: Group PM+ participants and other Key Informants (KIs) will be selected through convenience sampling. Informed consent will be obtained using a single step procedure where participants are provided oral and written information about the study and its purpose in the local language. The number of KI interviews in each category of respondent will be determined by empirical saturation, with a minimum of 2–16 participants per each category. FGDs will also be conducted in relevant categories.

#### Qualitative data analyses

The qualitative data collected from FGDs, key informant interviews, and notes during the process evaluation will be coded in NVIVO [60] and analyzed using content analysis [61] on the translated transcripts of the original language. Coding will be conducted by multiple independent raters, and inter-rater reliability will be calculated using Kappa scores.

### Ethical considerations

Throughout the different study phases participants in both arms will have access to mhGAP-trained health staff in the districts. When necessary they will be referred to a specialist for further assessment or management of severe psychiatric problems. If a participant experiences psychological problems after the project, they will be offered additional support.

All adverse events (AEs) and serious adverse events (SAEs) that are reported spontaneously by the participant or observed by either research or intervention staff will be recorded. All staff will be trained in the TPO Nepal Adverse Events Reporting Mechanism, which guides the process of reporting and supporting/referral in case of any adverse events.

All AEs and SAEs will be reported to a local independent Data Safety Management Committee (DSMC). The DSMC includes psychiatrists, non-governmental organization experts in psychosocial programs, and researchers and is established specifically for oversight of the trial and review of SEs and SAEs. The chair or a nominated person from the DSMC will review SAEs within 48 h, deciding if an SAE is likely related or unrelated to the intervention. The DSMC will review all AEs once a month. In both instances the committee will, where necessary, determine any appropriate action in respect of ongoing trial conduct (i.e., referral to specialized care). All changes in treatment resulting from AEs or SAEs will be reported to the DSMC in Nepal. TPO Nepal is responsible for data collection and storage and making data available to the DSMC, funders, and IRBs for audits when appropriate.

The project has been approved locally by the Nepal Health Research Council, Kathmandu, Nepal and by the WHO Ethical Review Committee (version 3; protocol ID 2817, October 25, 2018).

### Dissemination

Findings from the c-RCT will be published through various channels. In Nepal the results will be disseminated to key stakeholders, including district, provincial, and national government, through Nepali and English reports and presentations. Internationally, the findings will be published in academic journals and reports to the research funder (Office of US Disaster Foreign Assistance/USAID) and disseminated through the Mental Health Innovation Network (www.mhinnovation.net). For authorship eligibility we will comply with guidelines of the International Committee of Medical Journal Editors. Also, additional attention will be given to recommendations for equitable representation of researchers from LMIC for academic authorship [62]. After publication of the primary analyses, the data will be made publicly available to keep with transparency recommendations.

## Discussion

The described c-RCT on the effectiveness of Group PM+ in Nepal has been informed by a preceding formative work and a feasibility c-RCT with Group PM+ in Nepal [10]. It will contribute to the building evidence base for transdiagnostic psychological interventions delivered by non-specialists for people in communities affected by adversity. It builds upon the results and shown effectiveness of individual PM+ in Kenya [11] and Pakistan [63] and the first RCT on the effectiveness of Group PM+ has been successfully completed in Swat valley in Pakistan [13].

After individual PM+ has been found to be effective in Kenya and Pakistan, it was released for use by the WHO [2]. The intervention manual is now used in different settings all over the world, increasing access to an evidence-based intervention for people with mental health problems. If Group PM+ is effective in both Pakistan and Nepal, the Group PM+ manual will also be published and available on WHO’s website for free. This will give opportunity for further adaptation and implementation of the intervention in diverse settings with communities that are in need of better access to psychological interventions. The intervention can be adapted for other LMIC and humanitarian settings, but also in high income settings where brief transdiagnostic group interventions are lacking.

### Trial status

The trial is open and recruiting as of November 25, 2018 and will likely be completed by May 31, 2019. The protocol (version 3) was last verified 25 October 2018. Subsequent protocol modifications will be reported to funders, IRBs, and registered with ClinicalTrials.gov.

## Data Availability

Data sharing is not applicable to this article as no datasets were generated or analyzed during the current study. See protocol manuscript for details on planned data sharing for data generated from planned study.

## Abbreviations

AE: Adverse event
AUDIT: Alcohol Use Disorder Identification Test
c-RCT: Cluster randomized controlled trial
CI: Community informant
CIDT: Community Informant Detection Tool
CMD: Common mental disorders
CPSW: Community psychosocial workers
DSMC: Data Safety Management Committee
ENACT: Enhancing Assessment of Common Therapeutic Factors
EUC: Enhanced usual care
FGD: Focus group discussion
GHQ: General Health Questionnaire
IRGT: Individually randomized group treatment trial
LMIC: Low- and middle-income countries
mhGAP: Mental health Gap Action Programme
ODK: Open data kit
PM+: Problem Management Plus
PHCCs: Primary health care centers
PHQ: Patient Health Questionnaire
PRIME: Programme for Improving Mental Health Care
PTSD: Posttraumatic stress disorder
SAE: Serious adverse event
TPO: Transcultural Psychosocial Organization Nepal
WHO: World Health Organization
WHODAS: World Health Organization Disability Assessment Scale
